# Influence of E‐beam irradiation on microbiological and physicochemical properties and fatty acid profile of frozen duck meat

**DOI:** 10.1002/fsn3.1386

**Published:** 2020-01-13

**Authors:** Muhammad Sajid Arshad, Joong‐Ho Kwon, Rabia Shabir Ahmad, Kashif Ameer, Sheraz Ahmad, Yunhee Jo

**Affiliations:** ^1^ Institute of Home and Food Sciences Government College University Faisalabad Pakistan; ^2^ School of Food Science and Biotechnology Kyungpook National University Daegu Korea; ^3^ Institute of Food and Nutritional Sciences PMAS‐Arid Agriculture University Rawalpindi Pakistan; ^4^ Department of Food Science Faculty of Biosciences Cholistan University of Veterinary and Animal Sciences Bahawalpur Pakistan

**Keywords:** E‐nose, fatty acid, frozen duck meat, ionization radiation, microbiology, sensory evaluation

## Abstract

This study investigated the effect of different doses (0, 3, and 7 kGy) of e‐beam on the microbiological and physicochemical qualities and the profile of fatty acids of the frozen duck meat (FDM). Electron beam at the dose of 3 kGy showed more than 2 log and 1 log cycles of reduction in the total bacterial (TAB) and coliform counts (TCC), respectively. The results indicated an increase in the TBARS values (1.50 ± 0.02 mg MDA/kg), peroxide value (0.83 ± 0.04 meq peroxide/kg), and total volatile base nitrogen (1.31 ± 0.16 mg/100 ml), but no effect on the sensory parameters. Irradiation lowered the lightness (L*) (31.87 ± 0.98) and redness (a*) (11.04 ± 0.20) values but elevated the metmyoglobin content in FDM. In addition, irradiation had no effect on the benzopyrene content; however, a reduction was observed in the vitamin A (0.239 ± 0.015 µg/g) and vitamin E (1.847 ± 0.075 µg/g) contents of the FDM samples. There were no trans‐fatty acids present in the treated (irradiated) as well as the untreated (nonirradiated) meat samples (FDM), whereas the fatty acid content decreased in irradiated samples, in contrast with the nonirradiated control. Electronic nose clearly discriminated between the nonirradiated and irradiated FDM based on principal component analysis. It is concluded that the e‐beam successfully improved the microbial quality of FDM with slight changes in physicochemical properties, but without altering its sensory properties.

## Graphical abstract

This study investigated the effect of several doses (0–7 kGy) of e‐beam on the microbiological and physicochemical qualities and fatty acid profile of frozen duck meat (FDM). E‐beam at 3 kGy showed more than 2 log and 1 log cycles of reduction in the total bacterial and coliform counts, respectively. The results indicated an increase in the levels of thiobarbituric acid reactive substances, peroxide value, and total volatile base nitrogen, but no effect on the sensory parameters.

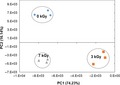

## 1 INTRODUCTION

Duck meat is famous worldwide, particularly in Asia, due to its attractive flavor, taste, nutritious value, and delicate texture (Chen et al., 2010; Khan et al., 2014). Duck meat contains high levels of polyunsaturated fatty acids, particularly linoleic and linolenic acid, compared with other meats. Due to its popularity, the consumers are more interested to enhance the safety of commercial frozen duck meat (FDM) (Heo et al., 2015; Kim et al., 2012; Kim, Lee, Lee, Choi, & Cho, 2011). Owing to its popularity, duck meat requires preservation using a technology that has minimal effects on the physiochemical properties of the meat. Irradiation is a very safe and effective method for food preservation as it minimizes spoilage and enhances the hygiene and shelf stability of food (Codex Alimentarius, 2003; WHO, 1994, 1999).

The use of irradiation for reducing the extent of microorganism contamination in food such as poultry, beef, fish, eggs, fresh fruits and vegetables, and spices are considered safe (Roberts, 2014). The maximum recommended doses for poultry, refrigerated meat, and frozen meat are 3.0, 4.5, and 7 kGy, respectively (ASTM, 2002; Kong et al., 2017). Different types of irradiation such as cobalt‐60 gamma rays, electron beam (e‐beam), and X‐rays are used for decontaminating food; however, the e‐beam irradiation is a better approach, which has the potential to minimize the problem of foodborne illnesses, and is a good alternative in the absence of thermal treatment. E‐beam irradiated foods are attractive for consumers because they are safer than the unirradiated foods. It also has a higher dose rate (10^3^–10^5^ Gy/s) as compared to gamma rays (0.01–1 Gy/s), and therefore, it requires less time for an equivalent level of decontamination (Li, Kundu, & Holley, 2015). E‐beam irradiation has the ability to enhance the microbiological safety and hygienic quality of FDM (An et al., 2018).

Ionizing radiation destroys microbial cells by demolishing essential macromolecules such as DNA, RNA, and proteins. For example, the electrons disrupt the DNA chain or change the position of the DNA molecule (Farkas, 2006; Samelis, Kakouri, Savvaidis, Riganakos, & Kontominas, 2005). This technique was used successfully for enhancing the shelf life of meat and meat products (Cabeza, Cambero, Hoz, & Ordóñez, 2007; Cabeza, Hoz, Velasco, Cambero, & Ordóñez, 2009; Selgas, García, & Calvo, 2009). However, the irradiation of meat may also cause an off‐odor and rancid flavor due to lipid oxidation in a dose‐dependent manner (Alfaia et al., 2007; Trindade, Mancini‐Filho, & Villavicencio, 2010). Oxidative stress may also affect myoglobin, which may lead to color deterioration (Duong et al., 2008).

The aim of the study was to evaluate the effect of different doses of e‐beam irradiation on the microbiological and physicochemical qualities of FDM, which includes comparison of fatty acid profile, electronic nose (e‐nose) pattern, and sensory characteristics between the nonirradiated and irradiated FDM samples.

## 2 MATERIALS AND METHODS

### 2.1 Materials and E‐beam irradiation

The samples of frozen meat of the duck or the FDM samples were purchased from a Korean retail store, Daegu (South Korea). The portion of the FDM containing fat was then taken out and separated, after that vacuum packaging was done before irradiation. The whole of the reagents and chemicals which were needed for conducting the present assessment were brought mainly through the well‐known companies named as Sigma Aldrich and Merck KGaA. The irradiation by e‐beam was performed by means of an efficient electron accelerator “(ELV‐4, 10 MeV, Fujifilm) at the EB‐Tech,” and by using an alanine‐electron paramagnetic resonance dosimetry system occupied with an “EMS 104 EPR analyzer” (Bruker Biospin), the absorbed doses of the radiation were fully estimated. In the current study, one control (nonirradiated) and two doses, that is, 3 and 7 kilo‐Gray, of the e‐beam irradiation were employed.

### 2.2 Proximate composition and microbiological analyses

The proximate composition of the FDM samples, which includes the significant levels of crude fat, crude protein, ash, carbohydrate, and moisture were calculated via the methods prescribed by the AOAC (2005) numbers 996.01, 992.15 (39.1.16), 920.153, 995.13 (30.1.23A), and 950.46B, respectively. The M.O. counts of the bacteria (TAB) and the coliforms (TCC) were enumerated by the procedures defined by AOAC, furthermore indicated as the colony forming units per gram (CFU/g). The estimated amount of the meat sample was then added to all of the enrichment broths and then incubated under optimal circumstances. Colonies isolated from each selective medium were identified using VITEK2 Compact (bioMerieux Ind.).

### 2.3 Physicochemical quality parameters

#### 2.3.1 Determination of pH and total volatile base nitrogen (TVB‐N)

The pH was determined in sample homogenates diluted with distilled water (1 → 10) using a digital pH meter (Orion 3 star, Thermoelectron Co.), which was calibrated with standard buffers of pH about 4.01, 7.00, and 10.01 at 25°C. The mean value was derived from three replicate measures of each experiment. The TVBN value of the irradiated FDM was calculated by using the microdiffusion procedure as described by Antonacopoulos and Vyncke (1989).

#### 2.3.2 Determination of the Hunter color value and heme pigment content

The FDM samples were tested in order to measure the value of surface color by means of an advanced colorimeter, that is, HunterLab (CR‐200; Minolta), having the measurements that were being standardized with reference to a white calibration‐plate (*L* = 89.2, *a* = 0.921 and *b* = 0.783). Thus, the values of color CIE *L* (lightness), CIE *a* (redness) and CIE *b* (yellowness) were gathered by making use of a standard value from eight random readings of the surface of each sample for the purpose of statistical analysis. In addition to this, relative concentration and/or amount of heme pigments, which include myoglobin (Mb), metmyoglobin (MMb), and oxymyoglobin (MbO_2_), were also determined according to Krzywicki (1982).

#### 2.3.3 Determination of TBARs and POV

By using the 2 thiobarbituric acid reactive substances method, known commonly as TBARS, by Ahn et al. (1998) along with a few modifications, the possible extent of the oxidation caused by lipids was estimated. By using a UV‐visible spectrophotometer (Optizen 2120UV; Mecasys Co. Ltd.) and at a wavelength of about 532 nm ain contrast to a blank solution containing aboout 1 ml and 2 ml distilled water and solution of TBA/TCA, respectively, the absorbance of the reaction resulting supernatant was calculated. The values of TBARS were elaborated as mg MDA/kg, that is, milligrams of malondialdehyde per kilogram of the meat. According to the method of the International Dairy Federation (IDF) peroxide value was also determined (Shantha & Decker, 1994). At 500 nm, the absorbance of the sample solution was estimated against a blank solution which contained all the reagents except the sample using a calibrated spectrophotometer. The unit meq peroxide/kg of meat was used to express the results.

#### 2.3.4 Determination of vitamins and benzopyrene

The determination of vitamin A and vitamin E from e‐beam‐irradiated FDM samples were determined by the method described by Thompson, Schimpf, and Baugh (2013). HPLC system (Agilent Technol.) was used for the determination of both vitamins. The flow rate 1.0 ml/min with column ID, 3 μm, 150 × 4.6 mm kept at 25°C was used. The benzopyrene content was determined by using the method described by Chen et al. (2012). HPLC system (Agilent Technol.) was used with C_18_ column (250 mm × 4.6 mm, 5 μm) kept at temperature 25°C and mobile phase consisted of acetonitrile and water with ratio of 9:1 at flow rate of 1.5 ml/min.

#### 2.3.5 The fatty acid profile determination

Lipids from the FDM samples were extracted by means of chloroform and methanol as put forward by Folch, Lees, and Sloane‐Stanley (1957). By bringing into use the technique of gas chromatography (Agilent, 6890) which was equipped with an advanced flame‐ionization detector, the methyl esters of fatty acid were being detected. Also, a fused‐silica capillary column (100 m × 0.25 mm × 0.2 µm; SP‐2560, Supelco) was made into use for the keen separation of the methyl esters of fatty acids. Fatty acids were identified by comparison of their retention times with those of a standard FAME mixture (SuplecoTM 37 Component FAME Mix, Catalog number 47885‐UP, Lot number LB‐85684; Sigma‐Aldrich Inc.). The fatty acid content was expressed as mg/g of total fatty acids identified and grouped as follows: saturated fatty acids (SFA), monounsaturated fatty acids (MUFA), polyunsaturated fatty acids (PUFA), and trans‐fatty acids.

#### 2.3.6 Determination of the e‐nose profile

The electronic or e‐nose (GC type E‐Nose; Heracles II, Alpha M.O.S. made in France) was thus used in order to monitor the “volatile flavor patterns” from e‐beam‐treated FDM samples. This instrument which was integrated with the classical GC functionalities in addition to an e‐nose olfactive fingerprint software, possesses an amazing capability of performing the complete and accurate analysis of data. Nearly, 1 g (gram) of the sample or less was placed in 10 ml headspace vials (22.5 × 75 mm, PTFE/silicon septum, aluminum cap). Five copies of each sample were orderly placed in the automatic sampler in the headspace system. The samples were heated to 40°C, and 1,000 μl of headspace gas from the gaskets was sampled with a syringe and injected into the equipment. The measurement phase lasted for 120 s and the clean phase lasted for 240 s.

#### 2.3.7 Sensory evaluation of the steamed FDM

The sensory evaluation of the e‐beam‐treated FDM was performed by 10 trained panelist considering a 9‐point hedonic scale (9 = like extremely; 1 = dislike extremely) based on the guidelines of Poste, Mackie, Butler, and Larmond (1991). The steaming of FDM was done in a special pan. Steaming is used as cooking method for meat. The scores of the sensory evaluation for several quality characteristics of the FDM just like, taste, texture, appearance, flavor, and palatability and its acceptability on the whole, were then noted accordingly. As for the evaluation process conduction is concerned, the respected panelists were served with crackers which were not salted, mineral water and expectorant cups in order to neutralize the taste and also to rinse their taste buds (receptors) for getting a rational assessment.

### 2.4 Statistical analysis

All the experiments were conducted in triplicates, excluding the Hunter color (HC) assay (8) and sensory evaluation (10). The data were expressed as mean ± standard deviation (*SD*) and were analyzed by using the one‐way analysis of variance (ANOVA) using the SPSS 19.0 software. The statistical significance value was precisely set to *p* < .05, which is the standard *p*‐value. The Duncan's multiple range (DMR) test was performed for the comparison of means.

## 3 RESULTS AND DISCUSSION

### 3.1 Proximate composition and microbiological qualities

The proximate composition of the e‐beam‐treated FDM samples is given here. Four parameters were determined for the chemical assay of FDM. The obtained results revealed the percentages of crude fat, ash and crude protein as well as moisture were estimated as 6.01 ± 0.099%, 0.97 ± 0.005%, 19.43 ± 0.481%, and 73.58 ± 0.149%, respectively. The results of the present study were concordant to the calculations done by Ali et al. (2007). He put out that the crude protein and ash contents in the meat of duck's breast were 20.06 ± 0.52% and 0.83 ± 0.11%, respectively. Qiao et al. (2017) demonstrated that the moisture and fat content in duck meat was 73.29 ± 0.42% and 5.92 ± 0.39%, respectively, which is similar to the findings of the present study. Furthermore, the protein and ash content were also similar to those reported by Michalczuk et al. (2016).

The microbial counts for total aerobic bacteria and coliforms in samples that were not exposed to the irradiation process and those of the irradiated samples of FDM are provided in Table 1. The microbial count of the nonirradiated samples for total aerobic bacteria and coliforms were 2.27 and 1.18 log CFU/g, respectively. A dose of 3 kGy completely decontaminated the total aerobic bacteria and coliform load from the FDM samples. The results of the present study corroborated the findings of Mahto, Ghosh, Das, and Das (2015), who demonstrated that the counts for total aerobic bacteria and coliforms were undetectable at and above 5 kGy. They also reported that the coliform count was sensitive and was eliminated at 1.5 kGy in frozen prawns, which is similar to our results where the total aerobic bacteria and coliform counts were negligible in the irradiated FDM samples. The previously obtained results were also explained and backed up with the help of other studies (Ahmed et al., 2009; Chouliara, Savvaidis, Panagiotakis, & Kontominas, 2004). Recently, Ham et al. (2017) demonstrated that doses of e‐beam like 2.5, 5, 7.5, and 10 kGy completely removed the total aerobic bacteria from cooked beef patties, which is in agreement with our findings.

**Table 1:** Effect of e‐beam irradiation on microbial loads, heme pigments, and Hunter's color of frozen duck meat

Parameter	Dose (kGy)		
	0	3	7
Microbial count (log CFU/g)			
Total bacteria	2.27	ND	ND
Coliforms	1.18	ND	ND
Heme pigment (%)			
Mb	42.52 ± 2.50a	41.5 ± 1.52a	42.89 ± 3.70a
OxyMb	16.42 ± 1.51a	15.07 ± 0.71a	12.4 ± 0.41b
MetMb	23.15 ± 0.77b	22.4 ± 1.85c	28.13 ± 5.02a
Hunter's color			
L*	34.06 ± 1.44a	32.26 ± 1.09b	31.87 ± 0.98b
a*	12.19 ± 0.63a	11.05 ± 0.62b	11.04 ± 0.20b
b*	5.88 ± 0.27b	6.12 ± 0.54ab	6.37 ± 0.30a

Note

Results are mean ± *SD* of the three independent determinations.

The different letters in a row represent significant differences (*p* < .05).

### 3.2 Heme pigment and Hunter color changes in FDM

“Myoglobin” is the heme protein responsible for meat color. The oxidation of iron atoms within the heme group (change of the red oxymyoglobin to the brown metmyoglobin) is responsible for the discoloration of meat. Oxygen is released when the ferrous heme iron is oxidized to the ferric form and is replaced by a water molecule (Faustman, Sun, Mancini, & Suman, 2010). The myoglobin, oxymyoglobin, and metmyoglobin's mean values of FDM, which was irradiated by using dissimilar doses of the electron beam are being shown in Table 1. Hence, the amounts of oxymyoglobin and metmyoglobin differed at large with the given doses, whereas the irradiation had no effect on myoglobin which is completely evident from the obtained results. Higher level of metmyoglobin was found in the FDM samples irradiated with 7 kGy, while the lowest or diminished oxymyoglobin and metmyoglobin levels were shown by the samples that were irradiated with 7 kGy and 3 kGy, respectively. The results showed that the amount of metmyoglobin significantly (*p* < .05) increased with the dosage of the irradiation. These results are also concordant to the estimations of Arshad et al. (2019), who reported that metmyoglobin contents significantly increased with the increase in the irradiation dose of 3 kGy in chicken meat. Furthermore, the results are supported by the outlines of An, Arshad, Jo, Chung, and Kwon (2017) who depicted that oxymyoglobin and metmyoglobin of the irradiated duck meat which was being smoked, increased significantly with the increase in the dose of irradiation (1.5–4.5 kGy).

The mean values for L* (lightness), a* (redness), and b* (yellowness) of FDM treated with the different e‐beam doses are given in Table 1. Results indicate that the values of L*, a*, and b* exhibited significant differences with the dose. Higher value for L* and a* were observed in the 0 kGy‐treated FDM, whereas higher value for b* was found in FDM treated with dose of 7 kGy (*p* < .05). The statistical results showed that minimum values for L* and a* were observed in FDM treated with both 3 and 7 kGy, whereas the minimum value for b* was found in 0 kGy‐treated FDM. The Hunter color values decreased slightly as the dosage of the e‐beam increased, except for the b* value. The results of the present study are consistent with those of Feng, Moon, Lee, and Ahn (2017), who showed that the L* and b* values of turkey breast meat decreased and increased, respectively, in a dose‐dependent manner. The degradation of water molecules by irradiation produces both oxidizing and reducing compounds (Thakur & Singh, 1994). These results were further supported by those of García‐Márquez, Cambero, Ordóñez, and Cabeza (2012). He reported that the redness and yellowness in the pork loin lowered and increased, respectively, with increase in the e‐beam dose. Giroux et al. (2001) demonstrated that the binding sites of free myoglobin react with hydroxyl or sulfur radicals produced by the irradiation of metmyoglobin and sulfmyoglobin. The irradiation produces a pigment similar to oxymyoglobin, where the myoglobin is primarily in the MnFe^3+^ form, which increases the a* value. In contrast, when the pigment is in the MbO_2_ form, irradiation converts the pigment into MbFe^3+^, which decreases the a* value (García‐Márquez et al., 2012). Recently, Ham et al. (2017) speculated that the decrease in redness of irradiated beef patties and pork sausages might be related to the disintegration of the nitrosyl hemochrome. The irradiation produced free radicals, which altered the color and enhanced the metmyoglobin content. The free radical generation could be more at higher doses.

### 3.3 Physicochemical quality of FDM

Lipid oxidation in meat and meat products starts at the time of slaughter and continues during storage. During irradiation, free radicals are produced, which cause chemical changes in meat such as lipid or protein oxidation (Kim et al., 2013). TVBN determines the degree of spoilage in meat due to bacterial growth and endogenous enzymes (Fan et al., 2009). Generally, 20 mg TVBN/100 g of beef is considered as the acceptable limit (Chen, Yang, Ou, Zhou, & Li, 2014).

The mean values of pH, TBARS, PO, and TVBN of FDM treated with dissimilar e‐beam doses are given in Table 2. It can therefore be clearly seen from the obtained results that the TBARS, POV, and the TVBN values varied effectively with the variation in dose, whereas pH showed no such effect. Higher TBARS, POV, and also TVB‐N were found in FDM irradiated with 7 kGy (*p* < .05), whereas minimum values for these parameters were observed in the nonirradiated FDM samples. Lipid oxidation increased with dose that is concordant to the results observed by Zhang, Wang, Zhang, Wang, and Ye (2016). He reported the fact that the TBARS values significantly (*p* < .05) increased in grass carp surimi with dose‐dependent manner (1–7 kGy). The increase in TBARS could be triggered by lipid oxidation, which is induced by the hydroxyl radicals produced by irradiation and carbonyl formation, disintegration of peroxides, and interaction with nucleophilic molecules (Aubourg, Perez‐Alonso, & Gallardo, 2004). Furthermore, Park et al. (2010) and Hocaoglu, Demirci, Gumus, and Demirci (2012) demonstrated that TBARS in beef sausages and shrimps increased with dose. Recently, Ham et al. (2017) reported that pH has no effect on meat quality (*p* > .05) at different doses and source, and TBARS values increased significantly (*p* < .05) in cooked beef patties and pork sausages after both gamma and e‐beam irradiation.

**Table 2:** Effect of e‐beam irradiation on physicochemical quality parameters of frozen duck meat

Parameter	Dose (kGy)		
	0	3	7
pH	6.22 ± 0.02a	6.19 ± 0.01a	6.23 ± 0.03a
TVBN (mg %)	0.75 ± 0.16b	0.93 ± 0.16b	1.31 ± 0.16a
TBARS (mg MDA/kg)	0.87 ± 0.03c	0.96 ± 0.06b	1.50 ± 0.02a
POV (meq peroxide/kg)	0.23 ± 0.02c	0.51 ± 0.02b	0.83 ± 0.04a
Vitamin A (µg/g)	0.383 ± 0.020a	0.278 ± 0.016b	0.239 ± 0.015c
Vitamin E (µg/g)	3.6 ± 0.148a	1.795 ± 0.307b	1.847 ± 0.075b
Benzopyrene (µg/g)	0.099 ± 0.012a	0.090 ± 0.001a	0.101 ± 0.013a

Note

Results are mean ± *SD* of the three independent determinations.

The different letters in a row represent significant differences (*p* < .05).

The mean values for vitamins A, vitamin E, and benzopyrene in FDM treated with different doses as given in the Table 2. The obtained results depicted that vitamins A and E have significant differences at different doses of radiation, whereas benzopyrene differed nonsignificantly among doses. Higher contents of vitamins A and E were detected in the nonirradiated samples, while minimum content of vitamins A and E were found in FDM treated with 7 kGy and 3 kGy radiation, respectively. Roberts (2014) reported that irradiation caused reduction in the vitamin content of various food produce. Our results also showed that irradiation significantly reduced the contents of vitamins A and E in FDM. Mohamed and El‐Deen (2016) demonstrated that the major degradable changes responsible for the loss of meat quality is lipid oxidation, which leads to the development of warmed‐off flavors, destruction of fatty acids, and vitamin loss. The results obtained were also in accordance with some recent findings done by Güler, Bostan, and Çon (2017). He speculated that the doses significantly reduced vitamin E content of food products. They also reported that irradiation‐induced vitamin E loss were higher in food products that were rich in fats.

### 3.4 Fatty acid profile of FDM

The mean values of fatty acids detected in the irradiated FDM are shown in Table 3. The fatty acid content differed significantly with dose. The levels of SFA, MUFA, PUFA, and trans‐fatty acids were determined. The results showed that the content of fatty acids decreased with increase in dose. Among the fatty acids, high levels were observed for cis‐9‐oleic acid in the nonirradiated FDM, followed by methyl palmitate. The lowest content among the 10 fatty acids was that of nervonic acid in the nonirradiated samples. Total SFA, MUFA, and PUFA levels were also higher in the nonirradiated samples, whereas reduction in the levels of total SFA, MUFA, and PUFA was observed in FDM irradiated with a 3‐kGy e‐beam. The FDM samples were estimated to contain no or negligible trans‐fatty acids. The fatty acid content decreased marginally with increase in dose. Zhang, Wang, Wang, and Ye (2017) showed that MUFA and PUFA levels in grass carp surimi decreased significantly with increase in the e‐beam dosage up to 7 kGy, which is consistent with the findings of the present study. Oxidative processes occur in unsaturated fatty acids due to the proximity of the carbon‐bonded hydrogen atoms to the double bonds, which lead to its replacement by higher reactive radical species produced by ionizing radiation (Brito, Villavicencio, & Mancini‐filho, 2002). Furthermore, Kim et al. (2011) also proposed the fact that unsaturated fatty acid contents decreased in the treated samples using 4 kGy radiation compared to that of the control, which is similar to our observation. The results are further supported by the findings of Nisar, Arshad, Yasin, Arshad, and Nadeem (2019) who found that fatty acid contents were slightly decreased by the irradiation treated chicken meat. The results are in accordance with the estimations done by Jo, An, Arshad, and Kwon (2018) who depicted that fatty acid contents were also decreased in with the use of e‐beam irradiation in the smoked meat of duck.

**Table 3:** Effect of e‐beam irradiation on fatty acid content (mg/g) of frozen duck meat

Fatty acids	Dose (kGy)		
	0	3	7
C_11:0_ 1	36.67 ± 0.41a	36.60 ± 0.51a	35.04 ± 2.72b
C_14:0_ 2	2.43 ± 0.03a	0.00 ± 0.00	1.84 ± 0.03b
C_15:0_ 3	3.83 ± 0.00c	4.97 ± 0.07b	5.81 ± 0.06a
C_16:0_ 4	80.50 ± 0.23a	71.06 ± 0.72b	69.37 ± 0.29b
C_16:1_ 5	12.11 ± 0.05a	8.43 ± 0.07b	8.82 ± 0.01b
C_18:0_ 6	32.09 ± 0.17b	34.73 ± 0.13a	35.58 ± 0.23a
C_18:1n9t_ 7	ND	ND	ND
C_18:1n9c_ 8	157.02 ± 0.23a	121.91 ± 0.23b	120.93 ± 0.77b
C_18:2n6t_ 9	ND	ND	ND
C_18:2n6c_ 10	68.04 ± 0.38a	58.15 ± 0.07b	57.75 ± 0.28b
C_20:4n6_ 11	20.60 ± 0.01c	24.64 ± 0.04b	29.23 ± 0.19a
C_24:1_ 12	0.07 ± 0.00b	0.12 ± 0.01a	0.07 ± 0.01b
Total	376.68 ± 0.28a	324.02 ± 1.20b	329.40 ± 1.85b
Ʃ SFA	118.85 ± 0.37a	110.76 ± 0.92b	112.61 ± 0.62b
Ʃ MUFA	12.18 ± 0.05a	8.55 ± 0.06b	8.89 ± 0.01b
Ʃ PUFA	245.66 ± 0.60a	204.71 ± 0.34b	207.91 ± 1.24b
Ʃ Trans fat	0.00	0.00	0.00

Note

Results are mean ± *SD* of the three independent determinations.

The different letters in a row represent significant differences (*p* < .05).

^1^ Methyl undecanoate.

^2^ Methyl myristate.

^3^ Methyl pentadecanoate.

^4^ Methyl palmitate.

^5^ Palmitoleic acid.

^6^ Methyl stearate.

^7^ Trans‐9‐oleic methyl ester.

^8^ Cis‐9‐oleic methyl ester.

^9^ Trans‐linoleic acid methyl ester.

^10^ Cis‐linoleic acid methyl ester.

^11^ Arachidonic acid methyl ester.

^12^ Nervonic acid methyl ester.

### 3.5 E‐nose profile of FDM upon e‐beam irradiation exposure

**Figure 1:**
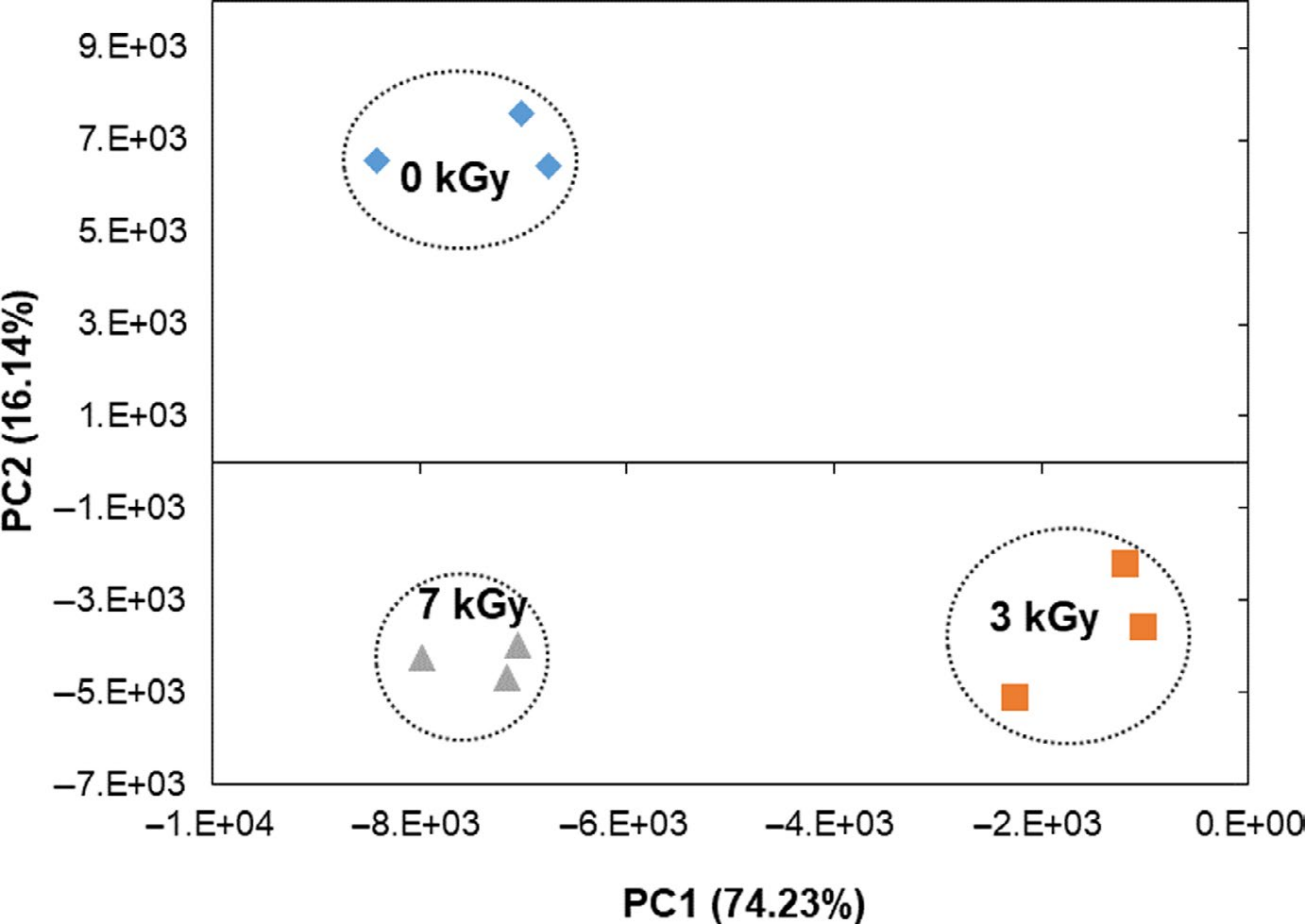
Effect of e‐beam irradiation on e‐nose profile of frozen duck meat based on PCA

E‐noses coupled with sensor technology are used for the detection of odors (Mendoza et al., 2014). It is an easy, rapid, accurate, and safe method for odor detection of samples, which underlines its usefulness in various applications (Wongchoosuk et al., 2014). The responses of the sensor to odor are nonlinear, and a technique similar to the principal component analysis commonly known as “PCA” was made into use. PCA was used for the elaboration of the e‐nose estimated results, with respect to nontreated and the treated FDM samples (Figure 1). The first principal component (PC1) was represented as 74.23%, while the second principal component (PC2) was denoted as 16.14%. The accumulative shares of PC1 and PC2 of the nonirradiated and irradiated FDMs were 90.37%. It is evident from Figure 1 that the nonirradiated and irradiated FDMs possessed distinct odors and were located in different corners of the PC plot. The samples that were not irradiated were displayed at the top most, left side of the PC plot while a 3‐kGy‐treated FDM was on the right corner; the 7‐kGy‐treated FDM was located at the left corner of the PC plot, opposite to the 0‐kGy‐treated FDM. The results obtained with the e‐nose were consistent with the estimations done by Ahn, Akram, Kwak, Jeong, & Kwon, 2013. He speculated the fact that e‐nose or electronic nose clearly discriminates between different doses on the basis of flavor pattern using PCA. Other researchers also supported that the e‐nose clearly distinguished between irradiated mushrooms on the basis of their volatile flavor profile (Akram, Ahn, Baek, Yoon, & Kwon, 2013). Furthermore, Kim, Ahn, Shahbaz, Park, and Kwon (2014) reported that e‐nose could differentiate between irradiated and nonirradiated food using PCA.

### 3.6 Sensory quality of FDM

**Figure 2:**
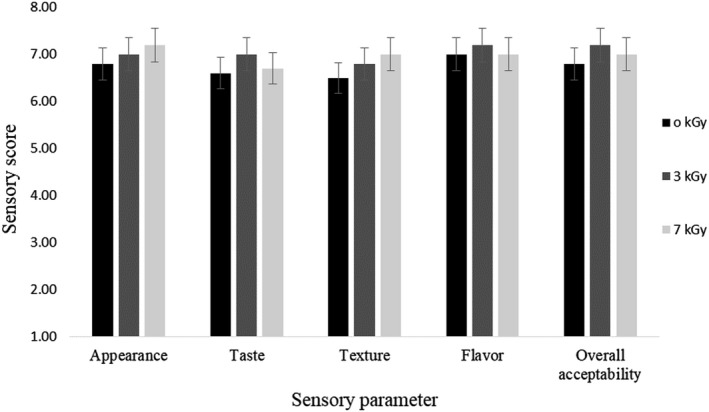
Effect of e‐beam irradiation on sensory parameter of frozen duck meat

The analysis of the sensory was embarked on various sensory quality characteristics and only if the score given by the worthy panelists was 5 or more than 5, the samples were therefore accepted “(Manju, Jose, Gopal, Ravishankar, & Lalitha, 2007).” The average or mean score of the sensory evaluation for all the parameters and the acceptability of irradiated FDM on the whole is given below in Figure 2. It is evident that the sensory parameters differed nonsignificantly with doses. Higher sensory score for appearance and texture was adjudged by the panelists to FDM treated with 7 kGy radiation; however, higher score was attributed to FDM treated with 3 kGy radiation for flavor, taste, and overall acceptability. The results showed that irradiation had no effect on the sensory parameters of FDM, which is in agreement with the findings of Al‐Bachir and Othman, (2013), who reported that chicken sausages treated with different doses (0, 2, 4, and 6 kGy) had no effect on the sensory properties. Vickers and Wang (2002) showed that irradiation had no effect on the acceptability of ground beef patties, and the sensory scores ranged from 6.1 to 6.4 on a 9‐point hedonic scale, which are consistent with the findings of the present study. Furthermore, Schilling et al. (2009) demonstrated that irradiation had no effect (*p* > .05) on the consumer acceptability of ground beef patties, and Fregonesi et al. (2014) showed that the sensory quality of irradiated lamb meat was unaltered, which corroborates our result.

## 4 CONCLUSIONS

The results showed that e‐beam has significant effect on the microbial quality, physic‐chemical parameters, and the profile of fatty acids of the frozen meat of duck. The total aerobic and coliforms counts in FDM were not detected with dose of 3 kGy and 7 kGy. The irradiated samples depicted slight increase in physicochemical parameters but this increase has no impact on the sensory attributes. The content of vitamin A and E decreased significantly in the irradiated samples whereas, no effect was observed in benzopyrene content. The saturated and polyunsaturated fatty acids were reduced in the irradiated samples however, there was no presence of fatty acids (trans) in both the irradiated as well as the nonirradiated frozen meat samples of duck. The principal component analysis for e‐nose clearly discriminated the treated (irradiated) and untreated (nonirradiated) frozen meat samples of duck. Conclusively, 3 kGy dose decontaminates the samples with minimum changes in physicochemical parameters with no effect on sensory attributes.

## CONFLICT OF INTEREST

The authors declare no conflict of interest.

## ETHICAL APPROVAL

This study has nothing to do with human and animal testing.

## DECLARATION

The authors did not use the human subjects.
